# Effects of experimentally-induced diabetes on sperm parameters and chromatin quality in mice

**Published:** 2013-01

**Authors:** Esmat Mangoli, Ali Reza Talebi, Morteza Anvari, Majid Pourentezari

**Affiliations:** 1 *Department of Biology and Anatomical Sciences, Faculty of Medicine, Shahid Sadoughi University of Medical Sciences, Yazd, Iran.*; 2 *Department of Andrology, Research and Clinical Center for Infertility, Yazd Reproductive Sciences Institute, Shahid Sadoughi University of Medical Sciences, Yazd, Iran.*

**Keywords:** *Sperm chromatin*, *Diabetes*, *Mice*

## Abstract

**Background: **Diabetes mellitus (DM), primary or idiopathic is a chronic disorder of the carbohydrate, lipid and protein metabolism. DM may impact male reproductive function at several levels. It is shown that DM has detrimental effects on sperm parameters in human and experimental animals.

**Objective:** The aim of this study was to observe the effects of diabetes on sperm parameters (viability, count, morphology and motility) and evaluation of sperm chromatin quality in mice.

**Materials and Methods: **Totally twenty adult male Syrian mice were divided randomly into 2 groups (n=10). The animals of group A were considered as controls while group B mice were diabetic that received a single dose (200 mg/kg) streptozotocin (STZ) intra peritoneally. After 35 days, the cauda epididymis of each diabetic mouse was dissected and placed in culture medium for 30 min. The swim-out spermatozoa were analyzed for count, motility, morphology and viability. The sperm chromatin quality and DNA integrity, was evaluated with Aniline Blue (AB), Toluidine blue (TB), Acridine orange (AO) and Chromomycin A3 (CMA3) staining.

**Results:** In sperm analysis, the diabetic mice had poor parameters in comparison with control animals (p=0.000). Regarding sperm chromatin quality, the results of TB and AO tests showed statically significant differences between two groups, but in AB and CMA3 staining, we didn’t see any differences between them.

**Conclusion:** The results showed that STZ-induced diabetes mellitus may influence the male fertility potential via affecting sperm parameters and DNA integrity in mice. However, according to our data, the diabetes doesn’t have any detrimental effects on histone-protamines replacement during the testicular phase of sperm chromatin packaging.

## Introduction

Diabetes mellitus (DM) is considered as one of the main stress to modern public health. Its rate is going up quickly and according to the World Health Organization (WHO, 2000) report, 177 million people were exaggerated by diabetes worldwide and by 2025; this figure will be raised to over 300 million (1). Factors such as obesity, population expansion and ageing are thought to be largely accountable (2). 

The large numbers of patients (90%) with type-1 diabetes are diagnosed before the age of 30. This type of diabetes is growing in European children by 3% annually, with a growing number being diagnosed in early childhood (2). As a consequence, DM will involve additional men prior to and during their reproductive years (3, 4). The occurrence of reproductive disorder in diabetic males is studied widely. Several experimental studies have demonstrated different kinds of male reproductive dysfunctions both structurally and physiologically in cases of DM (5, 6).

DM may affect male reproductive functions at multiple levels including its detrimental effects on endocrine control of spermatogenesis and/or by impairing erection and ejaculation (1). Ricc *et al* found that insulin-dependent diabetes is accompanied by reduced semen volume and decreased vitality and motility of the spermatozoa, but no change in seminal viscosity (6).

Another work from Queen's University has revealed that high level of blood sugar may affect sperm quality and therefore decreases male fertility potentials (3). There are some confirmations indicating higher rates of infertility in diabetic men and poor reproductive outcomes in comparison with healthy men (7). 

A new approach to the microscopic assessment of sperm for investigation of male fertility is the evaluation of sperm nuclear chromatin. Hence male gamete supplies 50 % of the embryonic genome, any anomalies in sperm chromatin can affect embryonic development. It is generally accepted that there is a clear relation between sperm chromatin/DNA damage and reproductive outcomes Furthermore sperm chromatin condensation has a key role in male fertility, early embryonic growth and pregnancy results (8-10).

In the process of spermatogenesis, the extent of sperm chromatin compaction changes deeply when histones are replaced at first by testis-specific nuclear proteins, then by transitional proteins and finally by protamines. Each abnormality during expression of sperm-specific nucleoproteins, changes sperm chromatin structure and may cause male infertility (8). The inter- and intra-molecular disulphide bonds of protamine molecules are crucial for sperm nuclear compaction and stabilisation. 

It is believed that this kind of nuclear compaction protects sperm genome from external damages include oxidative stress, temperature height and acid-induced DNA denaturation (11). There are many kinds of assays for the evaluation of sperm chromatin/ DNA which illustrate different forms of damages (9). Chromatin structural probes by nuclear dyes with cytochemical bases are sensitive, easy and inexpensive which do not require unique device like flow cytometry (8).

There are few studies that indicate the effects of DM on sperm DNA integrity. Agbaje *et al* measured the sperm DNA damage and found that the percentage of fragmented nuclear DNA and the number of deletions in mitochondrial DNA were significantly higher in specimen from diabetic men in comparison with non-diabetic ones (3). However, we couldn’t find any study indicating the relationship between sperm chromatin condensation and diabetes. 

Therefore, we designed this study to examine the possible relationship between sperm chromatin/DNA integrity using cytochemical assays, traditional sperm parameters and diabetes in mouse as an experimental model.

## Materials and methods

**Animals**

In this experimental study, totally 20 adult male Syrian mice (10 weeks old, 35g) were divided into 2 groups of A and B. The mice of group A considered as controls with no medication, whereas group B (diabetic mice) received diabetogenic agent, Streptozotocin (STZ) and basal diet. Both groups were housed in a controlled environment with a temperature range of 25±3^o^C and mean relative humidity of 50±5%. They were fed mice chow and had access to water ad libitum. This experimental project was approved by ethical committee of Shahid Sadoughi University of Medical Sciences. 

**Induction of diabetes**

In experimental group, the diabetes was induced via a single intra-peritoneal (i.p.) injection of buffered solution (0.1 mol/l of citrate, pH 4.5) of STZ at a dosage of 200 mg/kg body weight. At this dose, STZ induces considerable hyperglycemia (blood glucose > 250 mg/dl) in mice that was measured at 72 hours post-injection (4). After that, the blood glucose was measured periodically with the mean value 275 mg/dl in diabetic mice. 

**Epididymal sperm preparation**

After 35 days (one duration of spermatogenesis in mice is about 32 days), a small part of the cauda epididymis of each mouse was dissected and located in 1 mL of pre-warmed Hams F10 medium (37^o^C, 5% CO_2_). Gentle tearing of the tissue was done to make spermatozoa swim out into the culture medium. The dishes were placed in the incubator for 15 min. 

**Sperm analysis**

The sperm motility, normal morphology, viability and sperm count were evaluated for at least 200 spermatozoa of each animal. Sperm movement analysis was done by Makler Chamber and light microscopy (Olympus Co., Tokyo, Japan). Motility was expressed as the percentages of progressive motility including Rapid (Grade a) and Slow (Grade b) spermatozoa, non-progressive (Grade c) and immotile (Grade d) spermatozoa. The morphologically normal spermatozoa and the percentage of viable sprm cells were assessed by Papanicula staining and Eosin test respectively (10). The light microscope was set at ×40 eyepiece magnification. All analyses were performed by one experienced technician blinded to the study. Double checking of results was also done for each specimen. 

**Sperm chromatin/DNA evaluation**

DNA integrity and chromatin condensation assessments were assessed by standard cytochemical techniques including Acridine Orange Test (AOT), Aniline Blue (AB), Toluidine Blue (TB) and Chromomycin A3 (CMA3). All dyes and chemicals were purchased from Sigma Aldrich Company (St Louis,MO, USA). The efficacy of dyes was tested with and without acid denaturation of normal samples and they were considered as positive and negative controls, respectively (12).

**a. Aniline Blue (AB) staining**

Aniline blue selectively stains lysine-rich histones and is able to show those sperm chromatin condensation anomalies that are related to residual histones. To do this staining, air-dried smears from washed semen samples were fixed in 3% buffered glutaraldehyde in 0.2 M phosphate buffer (pH 7.2) for 30min at room temperature. Each smear was stained with 5% aqueous AB stain in 4% acetic acid (pH=3.5) for 7 min. In light microscopic evaluation, 200 spermatozoa were counted in different areas of each slide using ×100 eyepiece magnification (8).

**b. Toluidine Blue (TB) staining**

Toluidine blue is a metachromatic dye which shows both the quality and the quantity of sperm nuclear chromatin condensation/ DNA fragmentation via binding to phosphate groups of DNA strands (7). Briefly, air-dried sperm smears were fixed in fresh 96% ethanol and acetone (1:1) at 4^o^C for 30 min and then incubated in 0.1 NHCl at 4^o^C for 5 min. After that, the slides were washed 3 times with distilled water for 2 min and finally stained with 0.05% TB in 50% citrate phosphate for 10 min at room temperature. In each sample, at least 200 spermatozoa were counted under light microscopy with ×100 eyepiece magnification (13).

**c. Acridine Orange Test (AOT)**

Acridine orange is a metachromatic fluorescence probe for demonstration of degree of sperm nuclear DNA susceptibility to in-situ acid-induced denaturation by distinction between native double-stranded DNA (green fluorescent) and denatured single-stranded DNA (red fluorescent). Briefly, the air-dried smears were fixed in Carnoy’s solution (methanol/glacial acetic acid, 3:1) at 4^o^C for at least 2 hrs. Each sample was stained by freshly prepared AO (0.19 mg/ml in McIlvain phosphate-citrate buffer (pH=4) for 10 min. Smears were assessed on the same day using fluorescent microscope (Zeiss Co., Jena, Germany) with a 460-nm filter (8).

**d. Chromomycin A3 staining**

CMA3 is a fluorochrome specific for guanosine cytosine-rich sequences and it is used for estimate of the degree of sperm chromatin protamination (11). For this purpose, the smears were dried first and then fixed in Carnoy’s solution at 4^o^C for 10 min. The slide was treated with 150µl of CMA3 (0.25mg/ml) in McIlvain buffer for 20min. After staining in darkroom, the slides were washed in buffer and mounted with buffered glycerol. In each sample, at least 200 spermatozoa were counted under fluorescent microscope with a 460-nm filter and ×100 eyepiece magnifications (14).

**Statistical analysis**

Statistical analysis was performed by SPSS software version 18 for Windows (SPSS Inc., Chicago, IL, USA). Student’s *t*-test was applied to evaluate the data and the term ‘statistically significant’ was used to signify a two-sided p<0.001 for sperm parameters and cytochemical tests.

## Results

Table I shows the means and statistical analysis of the various sperm parameters in two groups. This table reveals that sperm count, rapid and total motilities, morphology and viability were significantly different (p=0.000) between Groups A and B. Table II shows the results of analysis of sperm chromatin and DNA integrity. 

Regarding to TB and AO tests, we saw significant differences (p=0.000) between two groups, but the results of CMA3 and AB staining didn’t show any differences between control and diabetic mice. It should be noted that in AB staining, the percentages of unstained or pale blue stained (normal spermatozoa) and dark blue stained (abnormal spermatozoa) were reported.

In TB staining, the chromatin quality of sperm was assessed according to metachromatic staining of sperm heads in following scores: 0, light blue (good chromatin); 1, dark blue (mild abnormal chromatin); 2, violet; and 3, purple (severe chromatin abnormality) (7). So, the sum of spermatozoa with score 1, score 2 and score 3 was considered as TB^+^ or sperm cells with abnormal chromatin, whereas score 0 spermatozoa were considered as TB^-^ or spermatozoa with normal chromatin. 

For AOT, the percentages of green (normal double-stranded DNA) and orange/red (abnormally denatured DNA) fluorescence spermatozoa per sample were calculated. In CMA3 staining, the bright yellow-stained reacted spermatozoa (CMA3^+^) were considered as abnormal form and yellowish green-stained non-reacted spermatozoa (CMA3^-^) were considered as normal form. 

**Table I.** The results of semen analysis in controls (group A) and diabetic mice (group B)

**Variables**	**Control mice (group A)** **Mean ± SD**	**Diabetic mice (group B)** **Mean ± SD**	p-value*
Count (×10^6^)	106.5±42.5	22.89±7.72	0.000
Rapid motility (%) (Grade a)	20.18±7.08	4.22±1.85	0.000
Slow motility (%) (Grade b)	22.64±5.0	16.22±6.05	0.002
Non progressive motility (%) (Grade c)	30.0±5.6	23.78±3.5	0.008
Immotile sperm (%) (Grade d)	27.09±9.98	55.7±4.68	0.000
Total motility (%) (Grade a,b,c)	72.91±9.98	44.22±4.68	0.000
Normal morphology	80.0±12.09	53.56±5.91	0.000
Viability (%)	75.09±9.42	47.56±6.22	0.000

Student’s t-test

**Table II:** The results of sperm chromatin/ DNA evaluation in controls (group A) and diabetic mice (group B)

**Variables**	**Control mice (group A)** **Mean ± SD**	**Diabetic mice (group B)** **Mean ± SD**	p-value*
TB	24.0 ± 9.96	48.33 ± 6.08	0.001
AO	9.82 ± 9.68	35.67 ± 6.44	0.001
CMA3	1.85 ± 1.34	3.14 ± 1.77	0.13
AB	13.14 ± 1.34	13.71 ± 2.05	0.7

Student’s t-test

**Figure 1:**
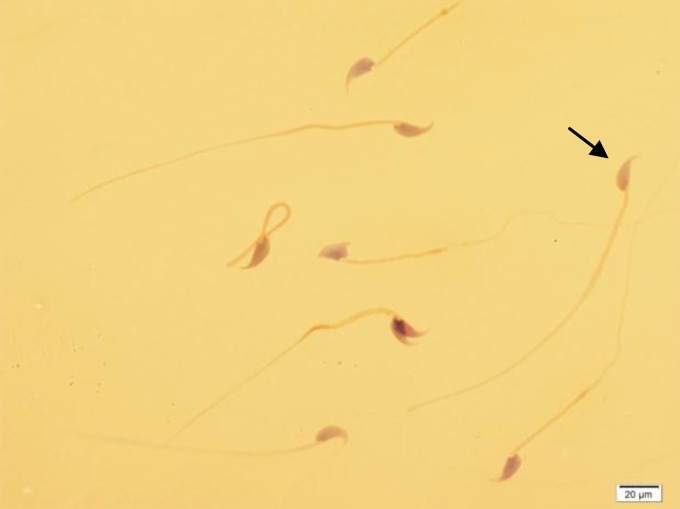
Different forms of sperm morphological abnormalities. The arrow indicates a normal spermatozoon. Papanicula staining, ×100 eyepiece magnification.

**Figure 2:**
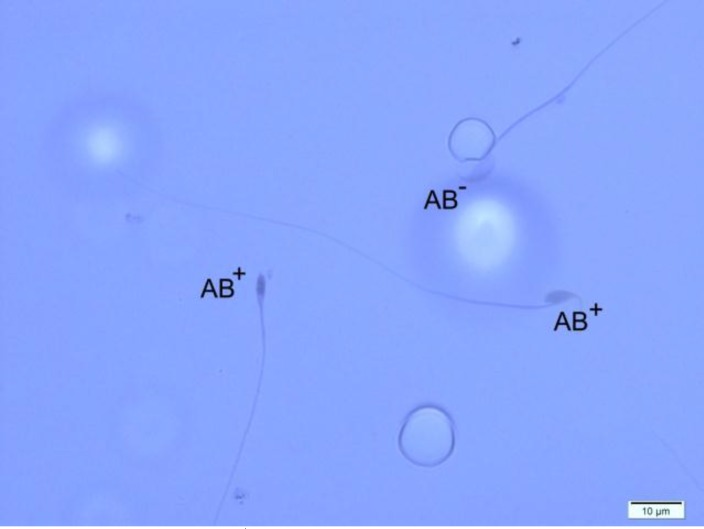
Two Aniline Blue-reacted spermatozoa (AB^+^) and one normal sperm cell (AB-).Aniline Blue staining, ×100 eyepiece magnification.

**Figure 3:**
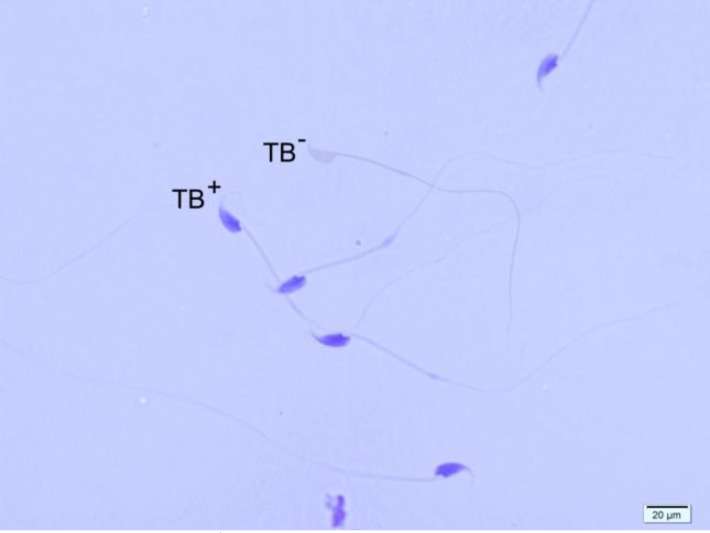
Toluidine Blue staining of spermatozoa. TB^+^ indicates sperm cells with abnormal chromatin and TB^-^ indicates sperm cells with normal chromatin (×100 eyepiece magnification).

**Figure 4:**
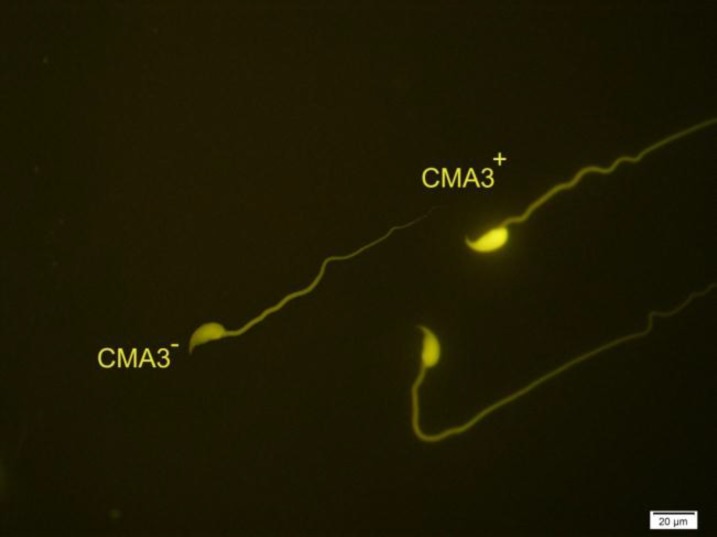
Two spermatozoa with protamine deficiency (CMA3^+^) and one sperm with normal protamine content (CMA3^-^). Chromomycin A3 staining, ×100 eyepiece magnification.

**Figure 5:**
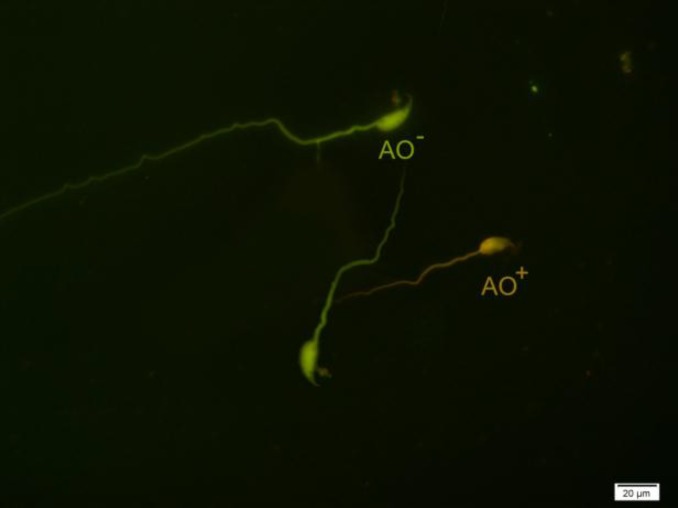
Spermatozoa with native double-stranded DNA (AO^-^) and denatured DNA (AO^+^). Acridine Orange staining, ×100 eyepiece magnification

## Discussion

Although it is suggested that DM may affect several parameters of male fertility like erection, semen volume, sperm count and testosterone level but the relationship between standard sperm parameters and diabetes is still controversial (15, 16). In present study, we assessed sperm quality and chromatin/ DNA integrity of spermatozoa in diabetic mice. All of the sperm parameters except slow motility had statistically significant differences between diabetic and control mice. 

The effect of DM on sperm parameters is a matter of debate. Petroianu *et al* didn’t see any differences in seminal concentration and rates of motile spermatozoa between diabetic and healthy men (1). Agbaje *et al* also examined spermatozoa from 27 diabetic and 29 non-diabetic men with the same average age. They found that even though semen volume in diabetic men was significantly lesser than controls (2.6 vs. 3.3 ml), there were no significant differences in sperm concentration, total sperm count, morphology and their ability to move between two groups (3). 

On the other hand, in verification of our results, Arikawe *et al* showed that in Alloxan-induced diabetic rats, all of the sperm parameters were significantly difference in comparison with controls (17). The differences in results may come from different kinds of species that were human and animals, and also different routes in diabetes induction. However, recently, Vignera *et al* in a brief review presented that clinical and experimental verification suggests that sperm parameters are affected in cases of DM. They also suggested that the involved mechanisms in the beginning of these alterations are hormonal changes, incidence of neuropathy, and enhanced oxidative stresses (18).

The other parameters which were compared between control and diabetic mice were sperm chromatin quality. It has been shown that the incidence of any anomaly in testicular expression and incorporation of every category of sperm-specific nucleoproteins may change sperm chromatin structure and will reduce male fertility potential (8). Although, there is few studies indicating the detrimental effects of diabetes on DNA integrity in human spermatozoa, but our study is the first report on the relationship between sperm chromatin quality and DM (3). Another unique feature of present work is using cytochemically-based dyes that demonstrate almost all of the problems that may occured in the process of testicular and epididymal phases of sperm chromatin remodelling.

Regarding TB staining, we found a significant difference between diabetic group and healthy control mice. This showed that DM may cause changes in both quality and quantity of nuclear chromatin condensation and increases the sperm DNA fragmentation. To compare our data with others, we didn’t see any similar study in the literature.

In AOT, we saw notable difference between two groups. As the AO has the potential to differentiate the single-stranded DNA from double-strand ones, it can be concluded that the DM increase the sperm DNA denaturation. It should be considered that this finding is confirmed by other researchers but with different assessment methods (3, 19). In CMA3 staining, although we saw a difference in percentage of CMA3-reacted spermatozoa between two groups, but it was not statistically significant. As we noted before, the CMA3 test shows the protamine deficiency in the process of sperm chromatin condensation (12). 

In AB staining that shows the sperm cells with excessive histones, we didn’t find any significant differences between two groups. So, considering AB and CMA3 results, we can say that the DM doesn’t have any detrimental effects on histone-protamines replacement during the testicular phase of sperm chromatin packaging. It is indicated that the level of oxidative stress is high in hyperglycemia state, due to excess production of reactive oxygen species (ROS) and decreased efficiency of anti-oxidant systems (20, 21). 

Oxidative stress is harmful to sperm and is considered as a main factor in male infertility (22). It is generally accepted that the oxidative stress impairs male fertility by changing the cell function like sperm motility, and increase in DNA damage by induction of gene mutations, DNA denaturation, base pair oxidation and DNA fragmentation (22-24). 

## Conclusion

In conclusion, our study showed that in the cases of diabetes, almost all of the sperm parameters had a statistically significant reduction in comparison with controls and also we demonstrated that spermatozoa of diabetic mice had less chromatin condensation and lower DNA integrity than spermatozoa of control group. Also, this study showed the importance of cytochemical evaluation of sperm chromatin and DNA integrity in cases of diabetes.
